# The Effects of a Novel Series of KTTKS Analogues on Cytotoxicity and Proteolytic Activity

**DOI:** 10.3390/molecules24203698

**Published:** 2019-10-15

**Authors:** Urszula Tałałaj, Paulina Uścinowicz, Irena Bruzgo, Arkadiusz Surażyński, Ilona Zaręba, Agnieszka Markowska

**Affiliations:** 1 Department of Organic Chemistry, Medical University of Białystok, Białystok 15-089, Poland; talalaj.urszula@gmail.com (U.T.); paulina.uscinowicz@umb.edu.pl (P.U.); irena.bruzgo@umb.edu.pl (I.B.); 2 Department of Medicinal Chemistry, Medical University of Białystok, Białystok 15-089, Poland; arkadiusz.surazynski@umb.edu.pl (A.S.); ilona.zareba@onet.eu (I.Z.)

**Keywords:** KTTKS, palmitoyl pentapeptide, plasmin inhibitors, cosmetic peptides, cytotoxity, collagen biosynthesis

## Abstract

KTTKS is a matrikine that originates from the proteolytic hydrolysis of collagen. This peptide stimulates ECM production and types I and III collagen expression in vitro. A more stable form of KTTKS is pal-KTTKS, known as Matrixyl^®^ or palmitoyl pentapeptide-3. A series of novel pentapeptides, analogues of KTTKS with the general formula X-KTTKS-OH(NH_2_), where X = acetyl, lipoyl, palmitoyl residues, was designed and synthesized. Their effect on amidolytic activity of urokinase, thrombin, trypsin, plasmin, *t*-PA, and kallikrein were tested. Cytotoxic tests on fibroblasts, as well as collagen and DNA biosynthesis tests for selected peptides, were also carried out. The test results showed that the most active plasmin inhibitors were palmitoyl peptides, whether in acid or amide form. No biological effects of lysine modification to arginine in the synthesized peptides were found. None of the synthesized peptides was not cytotoxic on fibroblasts, and three of them showed cell growth. These three compounds showed no concentration-activity relationship in the collagen and DNA biosynthesis assays.

## 1. Introduction

Aging is a natural process connected with decreased metabolic activity in cells and poor regenerative abilities of the human body [1,2]. Much effort is directed nowadays to the subject of skin aging largely due to the belief that a person’s image confirms their attractiveness and credibility. There are numerous products on the market, collectively referred to as “cosmeceuticals”, whose aim is to remedy skin aging processes such as dry skin or the appearance of wrinkles. The first anti-aging (anti-wrinkle) cosmeceuticals, introduced in the early 1990s, were fruit acids, which improve skin appearance through peeling and moisturizing. The next generations of anti-wrinkle products were retinol and its derivatives, used in the treatment of acne lesions. In addition, they smoothen wrinkles and help remove skin discolorations. The 21st century marks the appearance of lipopeptide technologies.

The concept of using peptides as cosmetics was based on a group of natural compounds called matrikines [3,4,5,6], whose name is a compound of the terms “extracellular matrix” + “cytokines”. An extracellular matrix (ECM) contains numerous extracellular proteins and glycosaminoglycans undergoing limited proteolysis, resulting in the release of biologically active fragments whose activity differs from that of full-length molecules [3,7]. This breakdown is caused by specific hydrolytic enzymes: Collagenases, elastases, hyaluronidases, or cathepsins. The process of hydrolysis in question is not accidental, as would have been the case with chemical acid hydrolysis, due to the release of peptides acting as endogenous regulators of many physiological and pathological processes [8]. Matrikines are mostly short, usually 3-8-amino acid long, but 700-amino-acid-long ones also do occur [9]. Examples of collagen-derived matrikines are endostatin, endotrophin, endorepellin, or tumstatin [10]. Peptides from the laminin α4 and α5 chains show antibacterial properties against *E. coli* and *S. aureus* [11]. Endostatin regulates adipose tissue weight in a mouse model of obesity [12]. Elastin peptides regulate insulin resistance in mice [13]. Endotrophin, a product of the breakdown of collagen VI released by adipocytes, may play a role in obesity-related cancers [14,15]. Endostatin and anastellin have the ability to form amyloid fibres and may contribute to the formation of amyloid deposits, causing neurodegenerative diseases [16,17].

The role of matrikines in the skin was described in 2005 by Tran [8]. ECM fragments such as hyaluronan oligosaccharides, laminin peptides A13 and C16, the LG4 domain of laminin, and tripeptide GHK regulate wound healing [18,19]. Tripeptide GHK itself, as well as its inclusion in collagen film preparations may stimulate wound healing through the increase of collagen synthesis by fibroblasts. Another peptide, i.e., peptide VVPQ derived from elastin, has a mitogenic effect on skin fibroblasts [20]. KKLRIKSKE-sequence peptides of the LG4 domain of laminin a3 contribute to epidermis repair as they induce adhesion and keratinocyte migration. Angiogenic peptides from the a1 (A13) chain and the c1 (C16) chain of laminin stimulate wound healing by stimulating fibroblast migration and reducing the expression of pro and active forms of MMP2 in fibroblasts [21,22]. Skin condition is also improved by hyaluronan fragments, depending on their size. The high molecular mass of hyaluronan (1000–1400 kDa) or its fragments with a low molecular mass (5–20 kDa) have no healing properties, whereas medium-sized fragments (100–300 kDa) enable faster wound closure [23,24].

KTTKS, lysine-threonine-threonine-lysine-serine is a matrikine originating from proteolytic hydrolysis of collagen [25,26]. This peptide stimulates ECM production as well as fibronectin and type I and III collagen expression *in vitro*. It also stabilizes mRNA, which enables TGF-β synthesis. A palmitoyl, a more stable form of KTTKS (pal-KTTKS), also known as Matrixyl^®^ or palmitoyl pentapeptide-3, is able to penetrate the stratum corneum and enhance collagen I production by fibroblasts in vitro [27]. Pal-KTTKS also significantly increases procollagen production and regulates hyaluronic acid synthesis by human fibroblasts in vitro. The clinical result of an oil-in-water moisturizing cream containing pal-KTTKS was assessed in a double-blind randomized placebo-controlled trial in 94 healthy Caucasian females with face wrinkles. After administration of pal-KTTKS twice daily for 8 and 12 weeks, the length of wrinkles, age spots, and skin texture were markedly improved and reduced in subjects using the cream containing pal-KTTKS compared to the placebo cream [25].

Skin aging is a natural, progressive process involving a reduction in its biological activity, a slowdown of its regenerative processes, and especially dryness [2,28,29]. Excessive activation of the system of plasminogen activation, i.e., a group of serine enzymes, was found in dry skin [30,31]. In normal skin plasmin is only found in the basement membrane of the epidermis, whereas in the case of dry skin, it is found throughout the whole epidermis [32,33]. Urokinase plasminogen activator (uPA) is also present in the stratum corneum of the epidermis, as confirmed in tests of fibrinolytic activity in the stratum corneum of the epidermis, which was counteracted by the addition of anti-urokinase antibody [34,35]. Urokinase activation in SC was confirmed in tests in vitro, in which a urokinase precursor (pro-uPA) became active after incubation with the insoluble component of homogenate SC. These results show that the urokinase-type plasminogen activator is activated in SC after breaking the barrier, which may activate the plasminogen/plasmin system in the epidermis [36]. A positive correlation between enzymes of the plasminogen system and transepidermal water loss was also found (TEWL) [37].

It has been shown that it is possible to inhibit the activity of the enzymes in question in the skin. A well-known antifibrinolytic, i.e., *trans*-4-(aminomethyl)cyclohexanecarboxylic acid (AMCHA, TXA), and its methylamide both have an inhibitory effect on plasmin, increasing the regeneration of the barrier function of the stratum corneum of the epidermis after tests with the use of the tape strippings method, exposed to acetone and dodecyl sulphate, preventing epidermis hyperplasia and alleviating conditions that cause skin dryness [38,39,40,41]. Unfortunately, fibrinolytic activity inhibition does not result in the inhibition of the active centre of plasmin or urokinase. AMCHA does not bind with the active site of plasmin, with kringle domains on plasmin and/or plasminogen, to be precise, thus preventing tPA/uPA-induced activation of plasminogen to plasmin. Voegeli et al. described a competitive and reversible plasmin and urokinase inhibitor, benzylsulfonyl-d-Ser-homoPhe- (4-amidinobenzylamide) (BSFAB), with the following values: K_i_ 29 nM for plasmin and 25 nM for urokinase [42]. Moreover, BSFAB was shown to be a selective inhibitor, as it did not inhibit other key proteases present in SC, such as tryptase, kallikrein 5 and 7, or neutrophil elastase. Kallikreins are enzymes necessary for peeling, which is why BSFAB does not have an impact on the process of peeling and does not induce dry skin.

By combining the application of cosmetic peptides in cosmetics for dry and aging skin with inhibited serine protease activity, enzymatic, cytotoxic, collagen, and DNA biosynthesis tests on fibroblasts in vitro of 30 new peptides were planned, synthesized, and performed. The synthesis was based on the following stem sequence: -Lys-Thr-Thr-Lys-Ser-. The purpose of the modifications introduced to the compound in question was to find the structure-activity relationship of plasmin and urokinase, i.e., the inhibitors. Moreover, 2 basic peptides were synthesized: KTTKS and pal-KTTKS, which constitute the standards used for the tests. N-terminal groups, i.e., acetyl, palmitic, and lipoic, were introduced into the molecules; certain peptide sequences were also modified by changing lysine into arginine. In order to obtain the designed peptides, chemical synthesis on a solid medium, also called solid-phase synthesis, was used. All the obtained peptides were characterized using retention time, while mass of the compounds was confirmed with the use of mass spectrometry.

Biological tests consisted of an assessment of antiamidolytic activity against enzymes from the serine protease group, i.e., plasmin, uPA (urokinase type plasminogen activator), thrombin, *t*-PA (tissue type plasminogen activator), trypsin, kallikrein, and chymotrypsin. In addition, the influence of all the obtained compounds on fibroblast survival, as well as selected peptides for collagen and DNA biosynthesis, was verified.

## 2. Results

### 2.1. Peptide Synthesis

Thirty new and two previously known (KTTKS-OH and Pal-KTTKS-OH) peptides were obtained with satisfactory yields, ranging from 42 to 68%, by solid phase peptide synthesis. Two polymer media were used for the synthesis. Peptides containing the acidic group were synthesized on a 2-chlorotrityl linker, whereas peptides in the form of amides– on a Rink Amide polymer with a 4-[(2,4-dimethoxyphenyl)(Fmoc-amino)methyl]phenoxyacetic linker. Introduction of N-terminal fragments of peptides was performed: with the palmitoyl chloride in the presence of a base method in the case of the palmitoyl group, the classical amino acid coupling method in the case of lipoic acid, and with 1-acetylimidazole in the case of acetyl residue. Peptides were purified by semi-preparative HPLC whereas the retention time shown in Table S1 was determined using an analytical column. Mass spectrometry confirmed mass identity in relation to the designed peptides. The sequences and physicochemical parameters of the synthesized peptides **1–32** are also presented in Table S1

### 2.2. Enzymatic Investigations

Peptides that demonstrate activity blocking enzymes cause inhibition of hydrolysis of the synthetic chromogenic substrate for the enzyme. This is observed in the form of a decrease in the concentration of *p*-nitroaniline released from the synthetic substrates. The tested compounds did not affect trypsin, kallikrein, *t*-PA, and chymotrypsin activity. The amidolytic activity results of plasmin, urokinase, and thrombin for the synthesized peptides are presented in Table 1.

**Figure 1:**
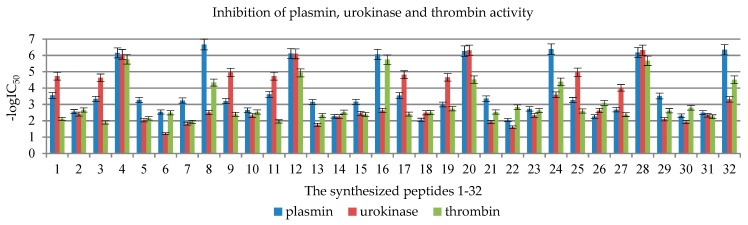
Inhibition of plasmin, urokinase, and thrombin by 1–32 peptides presented as -logIC_50_.

As IC_50_ values differ by several orders of magnitude, in order to present the results in the form of figures, they were converted into -logIC_50_. The values are presented in Table 2 and Figure 1.

Out of all the 32 designed compounds (Table 1 and Table 2 and Table S1, Figure 1), all N-palmitic peptides, i.e., **4**, **8**, **12**, **16**, **20**, **24**, **28**, and **32**, and both C-terminal acids and amides, turned out to be the most effective inhibitors of the tested enzymes. An analysis of the results shows that the most effective peptides that inhibit the activity of all three enzymes at a level above 90% are **20** Pal-Arg-Thr-Thr-Arg-Ser-OH and **28** Pal-Arg-Thr-Thr-Lys-Ser-OH.

In the case of plasmin, the greatest inhibition by palmitic peptides with micromole IC_50_ values was observed in the case of the most active peptide **8** Pal-KTTKS-NH_2_, 0.21 μM, to 0.89 μM for peptide **16** Pal-KTTRS-NH_2_ (Figure 1). The other peptides exhibited similar activity, with millimole IC_50_ inhibition values: from 0.24 mM in the case of Lip-KTTRS-OH (**11**) to 9.08 mM for Ac-RTTRS-NH_2_ (**22**, Table 1).

In the case of urokinase, peptides in the form of C-terminal acid turned out to be the most active. The most active were N-palmitic peptides Pal-Arg-Thr-Thr-Lys-Ser-OH (**4**), Pal-Lys-Thr-Thr-Arg-Ser-OH (**12**), Pal-Arg-Thr-Thr-Arg-Ser-OH (**20**), and Pal-Arg-Thr-Thr-Lys- Ser-OH (**28**, Figure 1). The calculated IC_50_ values for compounds **4**, **12**, **20**, and **28** are 0.00086, 0.0008, 0.00049, and 0.00048 mM, respectively (Table 1).

Thrombin activity inhibition was exhibited by N-palmitic peptides Pal-Lys-Thr-Thr-Arg- Ser-OH (**12**), Pal-Arg-Thr-Thr-Arg-Ser-OH (**20**), and Pal-Arg-Thr-Thr-Lys-Ser-OH (**28**). The calculated IC_50_ values for compounds **12**, **20**, and **28** are 0.012, 0.03, and 0,0021 mM, respectively (Table 1).

### 2.3. Viability of Fibroblast Cells

**Figure 2:**
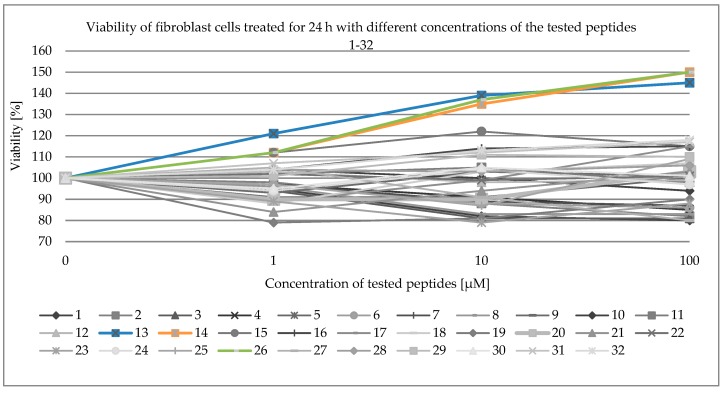
Viability of fibroblast cells treated for 24 h with different concentrations of the tested peptides 1–32 (Table 3).

Viability of fibroblast cells was examined with the use of the MTT assay. MTT, a yellow tetrazole, is reduced to purple formazan only in living cells. Absorbance of the coloured solution was measured by a spectrophotometer. The values are presented in Table 3 and Figure 2.

None of the synthesized compounds exhibited cytotoxicity towards fibroblasts (Figure 2 and Table 3). Peptides H-Lys-Thr-Thr-Arg-Ser-NH_2_ (**13**, blue line, Figure 2), Ac-Lys-Thr-Thr-Arg- Ser-NH_2_ (**14**, orange line, Figure 2), and Ac-Arg-Thr-Thr-Lys-Ser-OH (**26**, green line, Figure 2) exhibited cell growth effects.

### 2.4. DNA and Collagen Biosynthesis

Due to the fact that of those in the synthesized group, only peptides **13**, **14** and **26** showed proliferative effect on fibroblasts in the MTT test, only those compounds were investigated in DNA and collagen biosynthesis.

**Figure 3:**
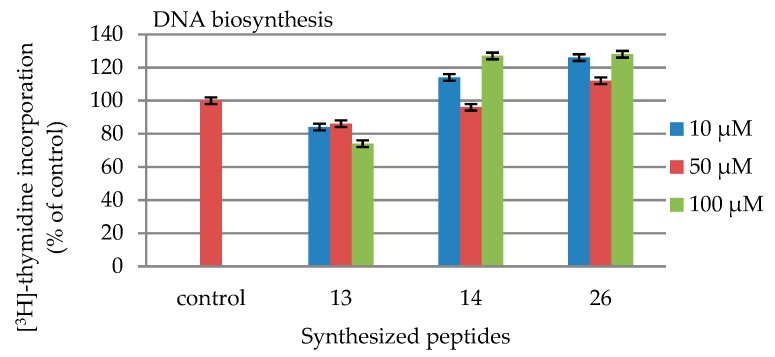
Influence of the synthesized peptides **13**, **14** and **26** on DNA biosynthesis.

In the radioactive method of DNA measurement in the liquid sample, the radioactive activity of the sample was measured. This technique involves mixing a radioactive material with a scintillation fluid/liquid (which contains a scintillator), resulting in photon emission. This emission is counted by a suitable detector. The values are presented in Table 4 and Figure 3.

**Figure 4:**
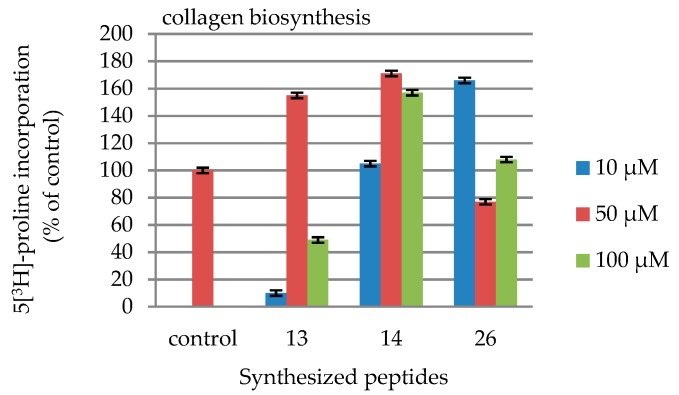
Influence of the synthetized peptides **13**, **14** and **26** on collagen biosynthesis.

In collagen biosynthesis assays cells were counted in a haemocytometer and incorporation of a radioactive precursor 5[^3^H]-proline into collagen was measured. Total protein synthesis was calculated from the sum of radioactivity of collagenase-resistant proteins and collagen digest. The values are presented in Table 5 and Figure 4.

None of the synthesized compounds exhibited cytotoxicity towards fibroblasts (Figure 2 and Table 3). Peptides H-Lys-Thr-Thr-Arg-Ser-NH_2_ (**13**, blue line, Figure 2), Ac-Lys-Thr-Thr-Arg- Ser-NH_2_ (**14**, orange line, Figure 2), and Ac-Arg-Thr-Thr-Lys-Ser-OH (**26**, green line, Figure 2) exhibited the effect of cell growth. In the case of these compounds, their influence of collagen (Table 5 and Figure 4) and DNA (Table 4 and Figure 3) biosynthesis were tested. In the case of DNA biosynthesis, no relationship with the concentration of the tested peptides was noticed; an increase in DNA synthesis was observed, however, in the case of compound **14**, both at the minimum concentration of 10 μM and at the maximum concentration of 100 μM. In the case of peptide **26**, increased DNA synthesis was observed at all concentrations. In the case of collagen biosynthesis, a significant increase was noticed, as high as up to approx. 160% for all the tested peptides, but there was no direct relationship with concentration.

## 3. Discussion

Human skin changes over time, throughout a person’s whole life. The formation of wrinkles is considered the most distinctive and the most common symptom of skin aging. Collagen is one of the most degrading proteins of the skin and its degradation is an important factor that affects skin aging. Degradation of collagen and other extracellular matrix (EM) components may also be associated with an increase in the damaging enzymes, such as plasmin and matrix metalloproteinase (MMP), which degrade laminin 332 and type IV and VII collagens [43].

The structure-activity relationships of 32 peptides in the following groups: C-terminal acids, C-terminal amides, peptides with a free N-terminal group, N-acetylated peptides, N-lipoic peptides, and N-palmitic peptides were analysed in order to consider the structural changes of the synthesized peptides as plasmin and urokinase inhibitors. The influence of lysine and arginine substitution in the basic KTTKS sequence was also analysed.

**Figure 5:**
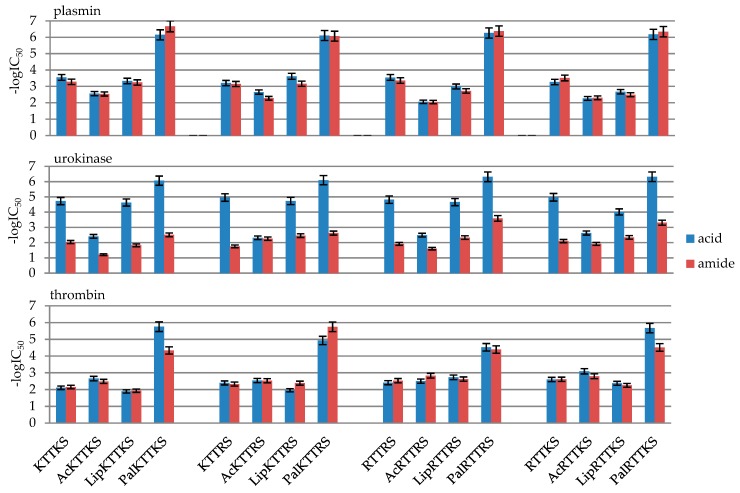
Influence of peptides in the form of C-terminal acids and amides on plasmin, urokinase and thrombin activity. Inhibition values in the form of -logIC_50_ from Table 3.

The first comparison was between the groups of C-terminal acids and peptide amides. Peptide activities were divided into sequences (Figure 5). The differences between the activity of acid and amide on plasmin were marginal. In each of the sequences, palmitic peptides exhibit the greatest exhibition of the enzyme. All palmitic acids and peptide amides exhibit IC_50_ values in the micromole range, as opposed to the other peptides, which exhibit inhibition values in the millimole range. In the case of free N-terminal peptides and their lipoic derivatives, the IC_50_ values are comparable. All acetylated peptides exhibit lower plasmin inhibition compared to other derivatives.

In the case of urokinase (Figure 5), it was noticed that amide peptides are weaker inhibitors compared to analogous acids. Peptides in the form of acids are the most active compounds not only towards urokinase, but also towards all the three enzymes described in this paper. In the case of the basic sequence H-Lys-Thr-Thr-Lys-Ser-OH v. H-Lys-Thr-Thr-Lys-Ser-NH_2_, IC_50_ is 0.019 mM for acid and 9.15 mM for amide (Table 1). As can be seen in Figure 3, a similar relationship is clearly visible also in the other cases. The relationship between acid-amide activity towards urokinase has previously been observed in the Department of Organic Chemistry, on the basis of inhibitors with a different sequence. A comparison was made between peptides in the form of C-terminal acid and amide with a sequence characteristic for urokinase inhibitors (Ac)H-D-Ser-Ala(Gly)-Arg-OH(NH_2_) [44]. Amides did not exhibit urokinase inhibition at the maximum examined concentration of 20 mM, despite the fact that they exhibited plasmin and thrombin inhibition.

In the case of thrombin exhibition, similarly to the case of plasmin, no significant differences in peptide activity resulting from the substitution of C-terminal carboxylic group for amide group were observed (Figure 5). All N-palmitic peptides are more active than others. Only in the case of a single peptide, whose sequence was Pal-KTTRS-NH_2_ (peptide **16**, Pal-Lys-Thr-Thr-Arg-Ser-NH_2;_ peptide **12**, Pal-Lys-Thr-Thr-Arg-Ser-OH), was greater amide activity towards acid noticed, i.e., IC_50_ was 0.0018 mM for amide **16**, 0.012 mM for acid **12** (Table 1).

**Figure 6:**
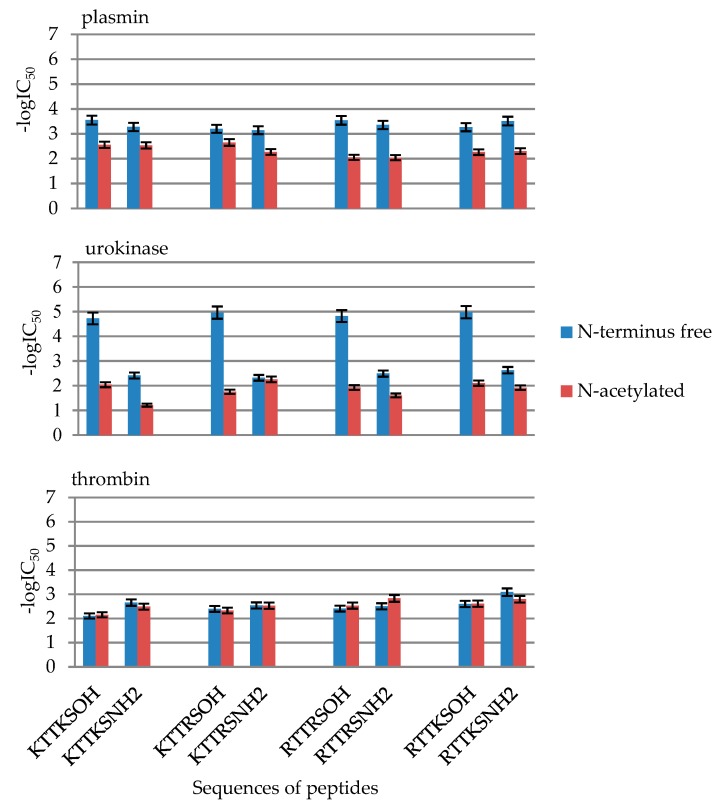
Influence of the introduction of N-terminal acetyl group in the synthetized peptides on plasmin, urokinase and thrombin inhibition. Inhibition values in the form of -logIC_50_ from Table 3.

In each of the eight sequences of the sequenced peptides, N-terminal acetyl group was introduced in order to test the influence of such a modification (Figure 6). The acetyl group (acetyl, Ac, -COMe, Lat. acetum) is an acetyl-type functional group derived from acetic acid. It occurs in its derivatives, e.g., acetyl chloride, acetic anhydride, acetate esters, or amides such as acetamide or acetanilide, among others. In organic synthesis it is used, e.g., as the protective group for amine and hydroxyl groups. In biological organisms acetyl groups are usually transferred from acetylo-CoA to other organic molecules [45]. Acetylo-CoA is also produced in the second stage of cellular respiration, i.e., the Krebs cycle, by the action of pyruvate dehydrogenase on pyruvic acid. Histones and other proteins are often modified through acetylation, which results in, e.g., changes in chromatin structure, making it possible for genetic transcription to occur. Acetylated organic molecules exhibit an increased ability to pass through the selectively permeable blood-brain barrier, which increases the efficacy of a given drug dose. The acetyl group in acetylsalicylic acid (aspirin) improves the drug’s efficacy compared to natural salicylic acid. Similarly, acetylation converts the natural analgesic morphine in the much stronger heroin (diacetylmorphine).

A very well-known molecule for cosmetic use containing the acetyl group, the N-terminal group in the case in question, is argireline [46]. Argireline is acetyl hexapeptide with the sequenceAc-Glu-Glu-Met-Gln-Arg-Arg-NH_2_. It is a fragment of SNAP-25 (which plays a role in the synaptic function of specific neuronal systems) and a substrate of botulinum 1 toxin. Argireline is used as a cosmetic ingredient to decrease the effects of aging by reducing wrinkles.

Acetylated peptides belong to the group of least active compounds of all those synthesized (Figure 6). Acetylated peptides at N-terminus are plasmin inhibitors with IC_50_ values from 2.24 to 9.08 mM (Table 1). The introduction of an acetylated fragment results in reduced inhibition of urokinase and thrombin activity.

Despite this clear evidence of increased activity, the synthesized N-acetylated peptides exhibit lower activity towards plasmin, urokinase, and thrombin than towards analogous sequences not containing the group in question. For, example, in the case of peptides H-Arg-Thr-Thr-Arg-Ser-OH (**17**) vs. Ac-Arg-Thr-Thr-Arg-Ser-OH (**18**), the highest reduction of activity towards plasmin was observed, with the following IC_50_ values: **17** N-free: 0.28 mM, while **18** N-acetylated: 8.81 mM (Table 1).

Lipoic acid (α-lipoic, LA) is an organic sulphur compound derived from octanoic acid which contains two sulphur atoms (in C6 and C8) bonded together with a disulphide bond and closed in a 5-membered ring. The carbon atom C6 is chiral, while the molecule occurs in the form of enantiomers (*R*)-(+)-lipoic acid (RLA), (*S*)-(-)-lipoic acid (SLA), and as a racemic mixture (*R/S*). In this paper, racemic acid was used. Lipoic acid is naturally produced in animals and plays a key role in oxidative metabolism [47,48,49,50]. It is a cofactor for at least five enzymatic systems. It also participates in the conversion of pyruvic acid to acetate and carbon dioxide and in the glycine cleavage system. Lipoic acid, as well as its reduced form, i.e., dihydrolipoic acid, may play a role in inactivation of free radicals. Moreover, they play a significant role in the regeneration of reduced forms of other antioxidants, such as vitamin C, vitamin E, glutathione, or ubiquinone. Lipoic acid has the ability to chelate metal ions (Fe^2+^, Cu^2+^_,_ Cd^2+^). It is also produced and available as a dietary supplement in certain countries, where it is sold as an antioxidant. Lipoic acid does not normally occur in the skin, but it can be a good candidate for topical use: it is a small, stable molecule that can penetrate the skin effectively and may protect from harmful effects of UV radiation; it is soluble both in water and in fats.

**Figure 7:**
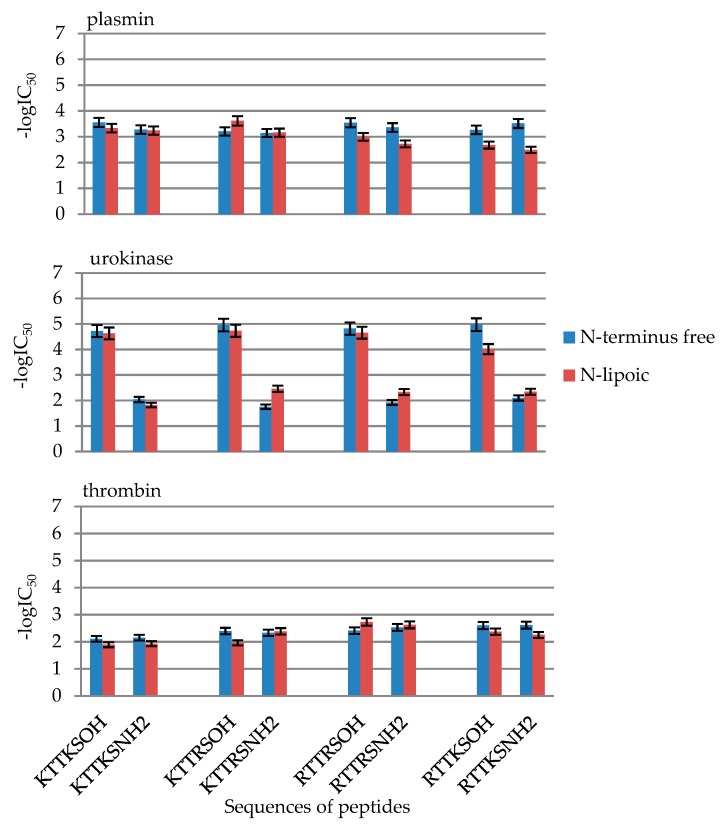
Influence of the introduction of N-terminal lipoic group in the synthetized peptides on plasmin, urokinase, thrombin inhibition. Inhibition values in the form of -logIC_50_ from Table 2.

In the case of plasmin inhibition, no significant differences were observed in the activity of N-free and N-lipoic peptides. In the case of RTTRS and RTTSK sequences, a decreased activity of lipoic peptides was observed, both by acid and amide. The introduction of lipoic residue resulted in increased activity towards plasmin only by KTTKS-OH; IC_50_ for N-free peptide H-Lys-Thr-Thr- Arg-Ser-OH (**9**) was 0.63 mM, whereas for lipoic peptide Lip-Lys-Thr-Thr-Arg-Ser-OH (**13**), the value was 0.24 mM. In the case of urokinase, three peptides exhibit activity higher than that of its N-free analogues: KTTRS-NH_2_ (**15**), RTTRS-NH_2_ (**23**) and RTTKS-NH_2_ (**31**). Owing to the fact that these are peptide amides, even this small increase of activity towards urokinase, compared to analogous acids, is uncommon (Figure 7).

The function of palmitic residue in cosmetics is softening, nutritional, and emulsifying [51]. Apart from the 16-carbon fragment, other residues of long-chain acids, such as myristic or lauric acid, are used. They are mostly surfactants, but their function in hair care products is also also play softening or antistatic. According to the information submitted to Food and Drug Administration (FDA) by the cosmetic industry in 2012 as part of Voluntary Cosmetic Registration Program (VCRP), over 20 palmitoyl oligopeptides, e.g.,: palmitoyl oligopeptide, palmitoyl dipeptide-7, palmitoyl tripeptide-3, palmitoyl tripeptyd-5, palmitoyl tripeptyd-8, palmitoyl-tripeptide-28, palmitoyl- tripeptide-38, palmitoyl-pentapeptide-4, or palmitoyl-hexapeptide-14 are in use [52,53].

The names of these peptides do not contain a particular sequence due to patent protection; it is, however, sometimes possible to obtain information on ingredient amino acids in literature data. For example, palmitoyl hexapeptide-36 contains, among others, aspartic acid, tryptophan, isoleucine, and phenylalanine. The palmitoyl peptides used in anti-aging creams are Pal-GHK and Pal-GQPR, ingredients of a preparation under the trade name Matrixyl 3000^®^. Palmitoyl pentapeptide-4 (palmitoil-pentapeptide-3 before 2006), on the other hand, whose sequence is KTTKS, studied in this paper, is a matrikine placed on the jest market in 2000, as an active ingredient of Matrixyl^®^, manufactured by the French producer of active cosmetics Sederma SAS (Le Perray en Yvelines Cedex, France).

**Figure 8:**
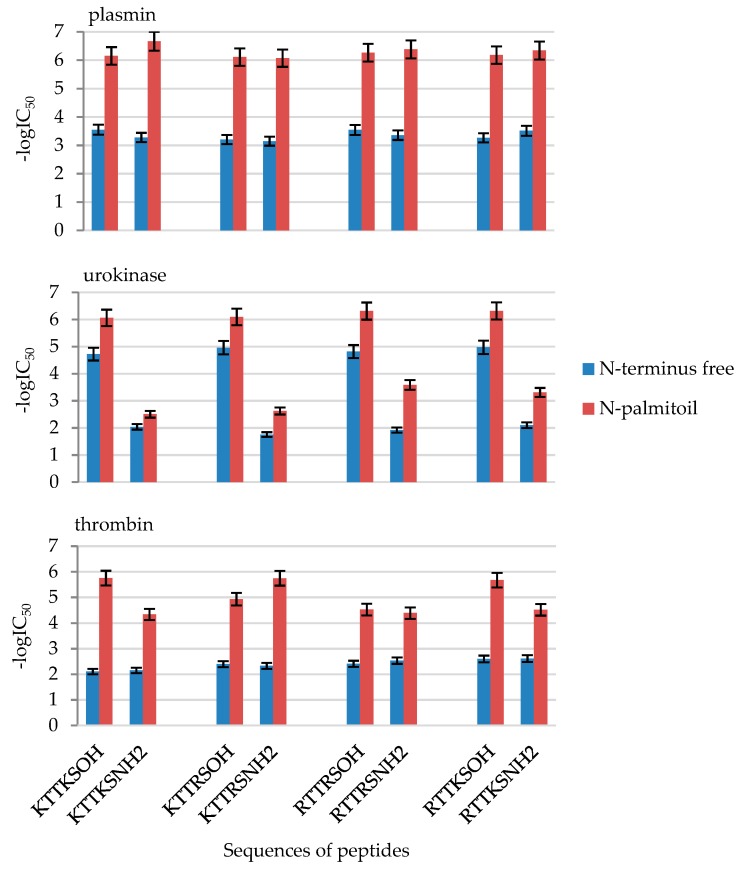
Influence of the introduction of N-terminal palmitic group in the synthetized peptides on plasmin, urokinase and thrombin inhibition. Inhibition values in the form of -logIC_50_ from Table 2.

Owing to the introduction of N-terminal palmitic group, the obtained peptides were the most active out of the whole series of 32 compounds (Figure 8). Each of the peptides exhibited increased activity towards all the enzymes, even the activity of amides towards urokinase increased. Peptide Pal-Lys-Thr-Thr-Lys-Ser-NH_2_ (**8**), inhibits plasmin activity at a concentration of 0.00021 mM. A peptide with such a high degree of enzyme exhibition has not yet been obtained in the Institute of Organic Chemistry. The lowest IC_50_ value of inhibition towards urokinase was exhibited by Pal-Arg-Thr-Thr-Lys-Ser-OH (**28**), 0.00048 mM, while the value of inhibition towards thrombin was 0.0018 mM for Pal-Lys-Thr-Thr-Arg-Ser-NH_2_ (**18**). Due to the presence of a long hydrocarbon chain, palmitic peptides should exhibit better skin permeability, in which case they could be used at lower concentrations but with higher activity.

In addition, modifications to the designed peptides included substitution of individual amino acids. A substitution of lysine for arginine was proposed, due to the fact that both amino acids are basic amino acids. These amino acids, however, differ in their structures as well as their roles in the body.

Arginine side chain consists of 3-carbon aliphatic straight chain ending in a guanidino group, (pKa 10.76), protonated and positively charged at physiological pH (pI 13.2). Due to the coupling between the double bond and the pairs of nitrogen atoms, the positive charge is delocalized, preventing the formation of hydrogen bonds. Lysine contains ε-amine group in the side chain, also in protonated form in biological conditions (pK_a_ 9.74; pI 10.28).

l-Arginine and l-lysine are amino acids which, apart from protein building, participate in many metabolic processes. In humans, l-arginine may be synthesized *de novo,* for example from glutamine, proline, and citrulline [54]. The amino acid is essential for the healthy growth and development of children, as well as adults in conditions displaying increased catabolism, trauma, and burns. It is a neurotransmitter in the central nervous system. l-lysine belongs to the group of exogenous amino acids, which means that the body should be supplied with it together with food, as humans are unable to synthesize it [55]. An increased demand for lysine occurs in the case of slow-healing wounds, osteoporosis, HSV infection, a strict slimming or vegetarian diet, and malnutrition. In the body, lysine and arginine use the same transport system. High lysine concentrations impair arginine absorption, and vice versa. Research shows that HSV has a large demand for arginine. High levels of lysine contributes to a decrease in arginine concentration, thus inhibiting HSV growth.

**Figure 9:**
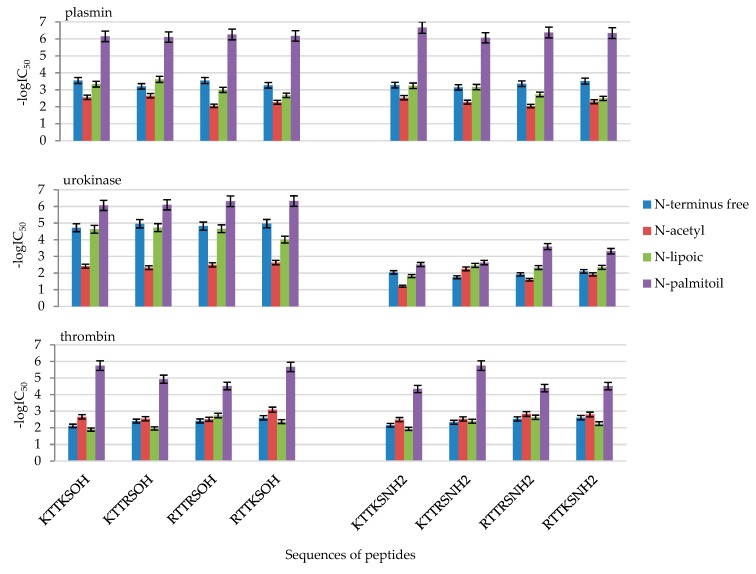
Influence of the synthetized peptides on plasmin, urokinase and thrombin activity with substitution of amino acids in sequences taken into account. Values in the form of -logIC_50_.

Substitution of lysine amino acids for arginine did not change the synthesized compounds’ activity towards plasmin. The general activity relationship in the basic KTTKS sequence was maintained. The introduction of N-terminal acetyl group resulted in decreased inhibition of plasmin activity by the synthesized peptides. The activity of peptides containing lipoic acid residue as the N-terminal fragment is comparable with peptides with a free amine group in the N-terminal amino acid. Despite the amino acid substitution, N-palmitic peptides, regardless of whether they are C-terminal acids or amides, are the most active plasmin inhibitors (Figure 9).

A similar relationship was observed in the case of urokinase inhibition (Figure 9). Substitution of amino acids did not change the general relationship, i.e., a decreased activity of acetylated peptides, a slight increase for lipoic peptides, with the highest activity of palmitic peptides maintained. Only in the case of KTTRS sequence was increased activity of acetylated and lipoic noticed compared to KTTKS sequence.

In comparison to the basic KTTKS sequence of acids, substitution of lysine for arginine slightly increased the activity in each of the cases, except palmitic basic sequence. In the case of amides, a similar relationship was noticed, i.e., increased activity, whereas in the case of KTTRS sequence, an identical value of IC_50_ was noticed, as was the case with the basic sequence.

In a similar our paper on the inhibition of plasmin and urokinase, the effect of amino acid substitution on a known inhibitor of similar sequence was described. In place of alanine in the sequence Ser-Ala-Arg other aliphatic [56] or aromatic [57] amino acids were introduced. In homoleucine and neoglycine, there was a loss of enzymes inhibition, but selectivity was found for α-methylalanine and α-aminobutanoic acid. These peptides were plasmin inhibitors, but lost their ability to inhibit urokinase. It would be more interesting if it was the other way around, because urokinase is a more selective enzyme. When alanine was replaced with aromatic amino acids, there was a loss of activity relative to urokinase.

No studies on amidolytic inhibition of proteolytic enzymes by cosmetic peptides are yet known. The only in vitro enzymatic study concerned the stability of KTTKS and pal-KTTKS in skin extracts and homogenates after addition of proteolytic enzyme inhibitors [58]. Pal-KTTKS retained in different skin layers (the stratum corneum, epidermis, and dermis) and KTTKS was not detected in any skin layer. Therefore, the authors confirm that only pal-KTTKS can be used as a cosmetic with anti-wrinkle effects.

The collagen production of human dermal fibroblasts under the influence of pal-KTTKS was described by Jonas et al. [27]. Palmitoyl peptide stimulates collagen production in a concentration-dependent manner while decreasing in the number of proliferative cells in the presents of conventional collagen stimulants and basal media.

Literature data concerning similar studies of collagen biosynthesis for KTTKS-OH, PalKTTKS-OH, LipKTTKS-OH, and KTTKS-vitamin C conjugate is available. In studies performed at in this paper: KTTKS-OH (**1**), PalKTTKS-OH (**4)** and LipKTTKS-OH (**3**) did not exhibit fibroblast growth, which is why they were not subjected to tests of their influence on collagen biosynthesis. Chichong Lu et al. described a significant, i.e., over 150%, increase of collagen biosynthesis in the case of LipKTTKS-OH [50]. Researchers tested peptides at a single concentration of 0.5 mM, considering a maximum concentration of 5 mM as non-toxic. In our research, three concentrations were used: 10, 50, and 100 μM, the same as in the case of cytotoxicity tests. In our study, LipKTTKS-OH did not influence fibroblast growth.

Another paper provides the results of tests of collagen synthesis for KTTKS-OH, PalKTTKS-OH, and ascorbic acid residue as N-terminal [59]. The authors report an over 150% increase of collagen synthesis in the case of PalKTTKS, but no such effect for stem KTTKS. An over 400% increase in the case of KTTKS-vitamin C conjugate was noticed at a concentration of 10 μM, and as high as over 650% at a concentration of 100 μM. In our case, the range of concentrations used was also 10-50-100 μM.

Plasminogen activation system causes damage of extracellular matrix and recovery can be enhanced by inhibiting these proteinases. Therefore, matrix components, including enzymes, are a good target for skin care products, because they can improve epidermal communication and skin homeostasis, thus strengthening the defence against “skin aging”. Despite the fact that we were unable to synthesize peptides with more favourable anti-aging properties than the parent KTTKS collagen fragment, this type of research should be continued due to the necessary search for new active cosmetic ingredients.

## 4. Materials and Methods

### 4.1. Materials

Rink amide resin, chloranil, acetaldehyde, HOBt = 1-hydroxybenzotriazole, lipoic acid, palmitoyl chloride, and 1-acetylimidazole were purchased from Fluka (Schnelldorf, Germany). 2-Chlorotrityl chloride resin, TFA = trifluoroacetic acid, DIPEA = diisopropylethylamine, piperidine, TBTU = tetrafluoroborate salt of the *O*-(benzotriazol-1-yl)-N,N,N,N’-tetramethyluronium tetra- fluoroborate, NMP = 1-methyl-2-pyrrolidon, Fmoc-Lys(Boc)-OH (Fmoc = 9-fluorenylmethyloxy- carbonyl, Boc = benzyloxycarbonyl) were obtained from Iris Biotech GmbH (Marktrewitz, Germany). Fmoc-Arg(Pbf)-OH, Fmoc-Ser(tBu)-OH and Fmoc-Thr(tBu)-OH were products of Lipopharm.pl (Gdańsk, Poland). DCM = dichloromethane, DMF = dimethylformamide, and methanol were the products of Chempur (PiekarySlaskie, Poland). DCM was used without further purification. DMF was distilled over ninhydrin and stored under molecular sieves 4A. HPLC solvent acetonitrile was purchased from Merck (Darmstadt, Germany). Urokinase, trypsin, kallikrein and Bzl-L-Arg-pNAHCl (Bzl = benzyl) were purchased from Sigma (Schnelldorf, Germany). Plasmin, S-2444 (pyro-Glu-Gly-Arg-pNA·HCl), S-2238 (H-D-Phe-Pip-Arg-pNA), S-2251 (H-D-Val-Leu- Lys-pNA), S-2266 (H-D-Val-Leu-Arg-pNA·2HCl and S-2288 (H-D-Ile-Pro-Arg-pNA) were obtained from Chromogenix (Milano, Italy). Thrombin and phosphate buffered saline (PBS) were purchased from Lubelska Wytwórnia Szczepionek (Lublin, Poland). *t*-PA was obtained from Boehringer Ingelheim GmbH (Ingelheim, Germany). Fibroblasts: Normal, Human, Adult ATCC^®^ PCS-201-012™ were purchased in ATCC, USA. Dimethylsulfoxide (DMSO), 3–(4,5-dimethylthiazol-2-yl)- 2,5-diphenyltetrazolium bromide (MTT) were purchased from Sigma Chemical Co. (St. Louis, MO, USA). Dulbecco’s minimal essential medium (DMEM), foetal bovine serum (FBS) were products of Gibco (San Diego, CA, USA). Glutamine, penicillin, and streptomycin were obtained from Quality Biologicals Inc. (Gaithersburg, MD, USA).

### 4.2. Peptide Synthesis

The peptides shown in Table S1 were synthesized manually using the standard Fmoc-based strategy [60]. Fmoc deprotection steps were performed with 20% (*v*/*v*) piperidine in DMF/NMP (1:1) for 3 and 8 min. separately. Peptide bonds with Fmoc amino acids were carried out through urea coupling reagent TBTU in DMF/NMP/DCM (1:1:1) of amino acid/TBTU/HOBt/resin using a molar ratio of 3:3:3:1. The reactions were monitored with the chloranil test. Cleavage from the resin was carried out with TFA/water (95/5). After 3 h stirring, the resin was filtered and washed with TFA. The combined filtrates were concentrated under reduced pressure. The crude peptide was washed with cold diethyl ether, filtered, dissolved in water and lyophilized. The Waters system (Waters Corporation, Milford, MA, USA) was used for analytical and semipreparatory HPLC (Phenomenex C18, Jupiter 90A, 4 micron, 250 × 4 mm; Phenomenex C18, Jupiter 300Å, 5 micron, 250 × 10 mm; solvents: A, 0.1% aqueous TFA; B, 0.1% TFA in acetonitrile, gradient 1% B to 99% B in A in 30 min, flow rate 1 mL/min, monitored at 220 nm). The major peak fraction was pooled and lyophilized. The molecular weight determination was performed by mass spectrometry using a Bruker Daltonics Esquire 6000 (Bruker Daltonik GmbH, Leipzig, Germany) with electrospray ionization (ESI).

### 4.3. Enzymatic Investigations

The determination of amidolytic activity was performed as previously described by Okada [61]. A detailed description of the method is given below: 0.2 mL of the examined peptides (0.15 M NaCl as contro), a buffer, and 0.1 mL of enzyme solution were mixed. The final peptide concentrations were: 0.2 mM, 2 mM, and 20 mM. The mixture was incubated at 37 °C for 3 min; then the synthetic substrate solution was added in the same buffer. After 20 min of incubation, the reaction was stopped by adding 0.1 mL of 50% acetic acid and the absorbance of the released *p*-nitroaniline was measured at 405 nm. The mean values from three independent experiments done in duplicate are presented. A method error of 5% was adopted as statistical error. IC_50_ value was considered as the concentration of inhibitor that reduces absorbance by 50%, compared with absorbance measured under the same conditions without an inhibitor. IC_50_ was calculated mathematically by extrapolation using a linear plot. IC_50_ values are presented in Table 1 and Table 2, and graphically in Figure 1.

The buffers and the enzyme solutions included:

- Tris buffer—0.6 mL (pH 8.8), enzyme: urokinase (50 units/mL), synthetic substrate: pyro-Glu-Gly-Arg-pNA·HCl (0.1 mL, 3 mM);
- Tris buffer—0.5 mL (pH 8.4), enzyme: thrombin (1 units/mL), synthetic substrate: H-D-Phe-Pip-Arg-pNA (0.2 mL, 0.75 mM);
- Tris buffer—0.5 mL (pH 7.4), enzyme: plasmin (0.4 units/mL), synthetic substrate: H-D-Val-Leu-Lys-pNA (0.2 mL, 3 mM);
- Borate buffer—0.5 mL (pH 7.5), enzyme: trypsin (0.4 units/mL), synthetic substrate: Bzl-L-Arg-pNA·HCl (0.2 mL, 8 mM);
- Tris buffer—0.6 mL (pH 9.0), enzyme: kallikrein (3 units/mL), synthetic substrate: H-D-Val-Leu-Arg-pNA·2HCl (0.1 mL, 75 mM);
- Tris buffer—0.6 mL (pH 8.4), enzyme: t-PA (167 mg/mL), synthetic substrate: H-D-Ile-Pro-Arg-pNA (0.1 mL, 10 mM)
- Tris buffer—0.6 mL (pH 9.0), enzyme: chymotrypsin (0.4 units/mL), synthetic substrate: Suc-Phe-pNA (0.2 mL, 8 mM)

### 4.4. Viability of Fibroblast Cells

#### 4.4.1. Cell Culture

These studies were performed on primary dermal fibroblasts cells. The cells were maintained in DMEM supplemented with 10% fetal bovine serum (FBS), 2 mM glutamin, 50 U/mL penicillin and 50 µm/mL streptomycin at 37 °C in a 5% CO_2_ incubator. For experiments, cells cultures were plated at a density of 1 × 10^6^/well in six-well culture plates (Costar, St. Louis, MO, USA) in 2 mL of growth medium. Cells reached about 80% of confluency at day 2 were used for the assays.

#### 4.4.2. Cell Viability Assay

Toxicity of the tested substances was determined using an MTT [3-(4,5-dimethylthiazol-2-yl)-2,5-diphenyltetrazolium bromide)] assay. Palmitoyl and lipoyl peptides were dissolved in DMSO (dimethyl sulfoxide), the other peptides in DMEM, and added to the cell culture medium. Simultaneously, the same concentrations of DMSO without peptides were added to cells, serving as controls. The final peptide concentrations were: 1 μM, 10 μM, and 100 μM. After 24 h incubation, the cells cultured with the tested compounds were washed with phosphate-buffered saline (PBS) and then incubated for 4 h in 2 mL of MTT solution (5 mg/mL). After removing the medium, the cells were lysed in 200 μL of DMSO with 20 μL of Sorensen’s buffer (pH 10.5, 0.1 mol/L glycine with 0.1 mol/L NaCl). The absorbance of converted dye in living cells was measured at a wavelength of 570 nm. The viability of the tested substances was calculated as a percentage of control cells and presented in Table 3 and Figure 2. The mean values ± SD from three independent experiments done in duplicate are presented.

#### 4.4.3. DNA Biosynthesis

To examine the effect of the studied compounds on DNA biosynthesis of fibroblast cells, they were plated in 24-well tissue culture dishes at 1 × 10^5^ cells per well with 1 mL of growth medium. The plates were incubated after 48 h with the examined peptides and 0.5 μCi of [^3^H] thymidine (6.7 Ci/mmol) at 37 °C. The final peptide concentrations were: 10 μM, 50 μM, and 100 μM. After 24 h incubation, the cells were rinsed with PBS three times and twice with 5% trichloroacetic acid. To measure the radioactivity incorporated into DNA, the cells were solubilized with 1 mL of 0.1 M sodium hydroxide containing 1% SDS and an addition of scintillation liquid (9 mL) (https://www.perkinelmer.com/pl/category/scintillation-cocktails). Radioactivity was determined in a scintillation counter, i.e., Liquid Scintillation Analyzer Tri-Carb 2810 TR (Perkin Elmer, Waltham, MA USA)) with Quanto Smart TM software (Perkin Elmer). The values are presented in Table 4.

#### 4.4.4. Collagen Biosynthesis

Fibroblasts were cultured in six-well plates until confluence. Then they were incubated for 24 h in growth medium in the absence or presence of the tested peptides and 5[^3^H]-proline (5 µCi/mL, 28 Ci/mM). The final peptide concentrations were: 10 μM, 50 μM and 100 μM. In accordance with the method proposed by Peterkofsky and Diegelmann [62], incorporation of the radioactive precursor into collagen was determined by digesting the proteins with purified *Clostridium histolyticum* collagenase. Finally, the monolayers were washed with sterile 10mM PBS, pH 7.4 four times, and cell membranes were disrupted using a sonicator. Aliquots of the cell homogenate were removed for protein measurement using the BCA™ Protein Assay Kit (Pierce, Waltham, MA USA). The results are shown as combined values for cell plus medium fractions. The values are presented in Table 5.

## 5. Conclusions

The purpose of the paper was to find the structure-activity relationship of novel analogues of KTTKS. The enzymatic activity of plasmin, urokinase and thrombin, the viability of fibroblasts, and DNA and collagen biosynthesis were evaluated. The results showed that the most active plasmin inhibitors were palmitoyl peptides. No effects of lysine modification on arginine in the synthesized peptides were found. Peptides showed no cytotoxicity on fibroblasts. Three of them did not show a correlation between the concentration increase in collagen biosynthesis and DNA.

## Figures and Tables

**Table 1:** Influence of the tested compounds on amidolytic activity of plasmin, urokinase, and thrombin in the form of IC_50_ [mM] (peptides most active as enzyme inhibitors are marked grey).

No	Peptide	IC_50_ [mM]		
		Plasmin	Urokinase	Thrombin
**1**	KTTKSOH	0.28 ± 0.014	0.019 ± 0.00094	7.81 ± 0.39
**2**	AcKTTKSOH	2.78 ± 0.14	3.88 ± 0.19	2.20 ± 0.11
**3**	LipKTTKSOH	0.47 ± 0.023	0.024 ± 0.0012	12.83 ± 0.64
**4**	PalKTTKSOH	0.00071 ± 0.000035	0.00086 ± 0.000043	0.0018 ± 0.00009
**5**	KTTKSNH_2_	0.53 ± 0.026	9.15 ± 0.46	7.04 ± 0.35
**6**	AcKTTKSNH_2_	2.92 ± 0.15	61.76 ± 3.09	3.25 ± 0.16
**7**	LipKTTKSNH_2_	0.58 ± 0.029	15.07 ± 0.75	11.70 ± 0.58
**8**	PalKTTKSNH_2_	0.00021 ± 0.000010	3.12 ± 0.16	0.046 ± 0.0023
**9**	KTTRSOH	0.63 ± 0.031	0.011 ± 0.00054	4.01 ± 0.20
**10**	AcKTTRSOH	2.24 ± 0.11	4.82 ± 0.041	2.89 ± 0.14
**11**	LipKTTRSOH	0.24 ± 0.012	0.019 ± 0.00093	10.94 ± 0.54
**12**	PalKTTRSOH	0.00078 ± 0.000039	0.00080 ± 0.00004	0.012 ± 0.00058
**13**	KTTRSNH_2_	0.72 ± 0.036	17.62 ± 0.88	4.65 ± 0.23265
**14**	AcKTTRSNH_2_	5.34 ± 0.27	5.56 ± 0.28	2.95 ± 0.15
**15**	LipKTTRSNH_2_	0.69 ± 0.035	3.47 ± 0.17	4.11 ± 0.21
**16**	PalKTTRSNH_2_	0.00086 ± 0.000043	2.37 ± 0.12	0.0018 ± 0.00009
**17**	RTTRSOH	0.28 ± 0.014	0.015 ± 0.00076	3.89 ± 0.19
**18**	AcRTTRSOH	8.81 ± 0.44	3.24 ± 0.16	3.12 ± 0.16
**19**	LipRTTRSOH	1.014 ± 0.051	0.022 ± 0.0011	1.84 ± 0.092
**20**	PalRTTRSOH	0.00055 ± 0.000027	0.00049 ± 0.000024	0.03004 ± 0.0015
**21**	RTTRSNH_2_	0.44 ± 0.022	11.97 ± 0.59	2.93 ± 0.14
**22**	AcRTTRSNH_2_	9.08 ± 0.45	24.96 ± 1.25	1.47 ± 0.074
**23**	LipRTTRSNH_2_	1.89 ± 0.094	4.67 ± 0.23	2.36 ± 0.12
**24**	PalRTTRSNH_2_	0.00042 ± 0.000021	0.26 ± 0.013	0.041 ± 0.0020
**25**	RTTKSOH	0.54 ± 0.027	0.0106 ± 0.00053	2.51 ± 0.13
**26**	AcRTTKSOH	5.45 ± 0.27	2.36 ± 0.12	0.81 ± 0.041
**27**	LipRTTKSOH	2.12 ± 0.11	0.097 ± 0.0049	4.23 ± 0.12
**28**	PalRTTKSOH	0.00067 ± 0.000033	0.00048 ± 0.000024	0.0021 ± 0.00011
**29**	RTTKSNH_2_	0.30 ± 0.015	7.95 ± 0.40	2.43 ± 0.12
**30**	AcRTTKSNH_2_	5.00 ± 0.25	12.07 ± 0.60	1.59 ± 0.080
**31**	LipRTTKSNH_2_	3.23 ± 0.16	4.57 ± 0.23	5.59 ± 0.28
**32**	PalRTTKSNH_2_	0.00045 ± 0.000023	0.49 ± 0.024	0.0306 ± 0.0015

**Table 2:** Influence of the tested compounds on amidolytic activity of plasmin, urokinase, and thrombin in the form -logIC_50_ (peptides most active as enzyme inhibitors are marked grey).

No	Compound	-logIC_50_		
		Plasmin	Urokinase	Thrombin
**1**	KTTKSOH	3.55 ± 0.18	4.72 ± 0.24	2.11 ± 0.11
**2**	AcKTTKSOH	2.56 ± 0.13	2.41 ± 0.12	2.66 ± 0.13
**3**	LipKTTKSOH	3.33 ± 0.17	4.63 ± 0.23	1.89 ± 0.09
**4**	PalKTTKSOH	6.15 ± 0.31	6.06 ± 0.30	5.75 ± 0.29
**5**	KTTKSNH_2_	3.28 ± 0.16	2.04 ± 0.10	2.15 ± 0.11
**6**	AcKTTKSNH_2_	2.53 ± 0.13	1.21 ± 0.06	2.49 ± 0.12
**7**	LipKTTKSNH_2_	3.24 ± 0.16	1.82 ± 0.09	1.93 ± 0.10
**8**	PalKTTKSNH_2_	6.67 ± 0.33	2.51 ± 0.13	4.34 ± 0.22
**9**	KTTRSOH	3.20 ± 0.16	4.96 ± 0.25	2.40 ± 0.12
**10**	AcKTTRSOH	2.65 ± 0.13	2.32 ± 0.12	2.54 ± 0.13
**11**	LipKTTRSOH	3.61 ± 0.18	4.73 ± 0.24	1.96 ± 0.10
**12**	PalKTTRSOH	6.11 ± 0.31	6.10 ± 0.30	4.93 ± 0.25
**13**	KTTRSNH_2_	3.15 ± 0.16	1.75 ± 0.09	2.33 ± 0.12
**14**	AcKTTRSNH_2_	2.27 ± 0.11	2.25 ± 0.11	2.53 ± 0.13
**15**	LipKTTRSNH_2_	3.16 ± 0.16	2.46 ± 0.12	2.39 ± 0.12
**16**	PalKTTRSNH_2_	6.07 ± 0.30	2.63 ± 0.13	5.75 ± 0.29
**17**	RTTRSOH	3.54 ± 0.18	4.82 ± 0.24	2.41 ± 0.12
**18**	AcRTTRSOH	2.05 ± 0.10	2.49 ± 0.12	2.51 ± 0.13
**19**	LipRTTRSOH	2.99 ± 0.15	4.66 ± 0.23	2.74 ± 0.14
**20**	PalRTTRSOH	6.26 ± 0.31	6.31 ± 0.32	4.52 ± 0.23
**21**	RTTRSNH_2_	3.36 ± 0.17	1.92 ± 0.10	2.53 ± 0.13
**22**	AcRTTRSNH_2_	2.04 ± 0.10	1.60 ± 0.08	2.83 ± 0.14
**23**	LipRTTRSNH_2_	2.72 ± 0.14	2.33 ± 0.12	2.63 ± 0.13
**24**	PalRTTRSNH_2_	6.38 ± 0.32	3.59 ± 0.18	4.39 ± 0.22
**25**	RTTKSOH	3.27 ± 0.16	4.97 ± 0.25	2.60 ± 0.13
**26**	AcRTTKSOH	2.26 ± 0.11	2.63 ± 0.13	3.09 ± 0.15
**27**	LipRTTKSOH	2.67 ± 0.13	4.01 ± 0.20	2.37 ± 0.12
**28**	PalRTTKSOH	6.18 ± 0.31	6.32 ± 0.32	5.67 ± 0.28
**29**	RTTKSNH_2_	3.51 ± 0.18	2.10 ± 0.10	2.61 ± 0.13
**30**	AcRTTKSNH_2_	2.30 ± 0.12	1.92 ± 0.10	2.80 ± 0.14
**31**	LipRTTKSNH_2_	2.49 ± 0.12	2.34 ± 0.12	2.25 ± 0.11
**32**	PalRTTKSNH_2_	6.34 ± 0.32	3.31 ± 0.17	4.51 ± 0.23

**Table 3:** Viability of fibroblast cells treated for 24 h with different concentrations of the tested peptides 1–32 (% of Control ± 2). Peptides exhibiting a significant cell growth effect are marked grey.

**Synthesized Peptides**																
**Concentration** **[µmol/L]**	**1**	**2**	**3**	**4**	**5**	**6**	**7**	**8**	**9**	**10**	**11**	**12**	**13**	**14**	**15**	**16**
1	95	98	94	97	91	97	97	96	93	104	102	103	121	112	112	104
10	82	88	81	91	91	88	89	83	103	100	105	98	139	135	122	114
100	80	82	81	85	100	86	87	83	101	94	98	99	145	150	115	115
**Synthesized Peptides**																
**Concentration** **[µmol/L]**	**17**	**18**	**19**	**20**	**21**	**22**	**23**	**24**	**25**	**26**	**27**	**28**	**29**	**30**	**31**	**32**
1	89	88	79	90	84	102	89	94	97	112	105	104	101	95	107	104
10	99	103	81	90	94	99	79	104	87	137	91	103	111	105	112	113
100	115	107	90	103	103	101	88	97	109	150	80	106	110	101	117	118

Control without peptides, viability 100%.

**Table 4:** DNA biosynthesis (% of Control ± 2).

	Peptides		
Concentration [µmol/L]	13	14	26
10	84	114	126
50	86	96	112
100	74	127	128

**Table 5:** Collagen biosynthesis (% of Control ± 2).

	Peptides		
Concentration [µmol/L]	13	14	26
10	10	105	166
50	155	171	77
100	49	157	108

## Supplementary Materials

The following are available online, Table S1: Sequences and physico-chemical parameters of synthesized peptides 1–32.
