# Supplementary material for: The Effects of a Novel Series of KTTKS Analogues on Cytotoxicity and Proteolytic Activity
Source: Molecules. 2019 Oct 15;24(20):3698. doi: 10.3390/molecules24203698 (PMC6832239; doi:10.3390/molecules24203698)
Supplement: Supplementary file 1 [file molecules-24-03698-s001.pdf]

Suplementary file

The effects of a novel series of KTTKS analogues on cytotoxicity and proteolytic activity

Urszula Tałałaj[1], Paulina Uścinowicz[1], Irena Bruzgo[1], Arkadiusz Surażyński[2], Ilona Zaręba[2], Agnieszka Markowska[1].

Table 1. Sequences and physico-chemical parameters of synthesized peptides 1-32.

No	Sequences of the synthesized peptides		Yield [%]	Retention time [min]	MW	[M+H]+
1	H-Lys-Thr-Thr-Lys-Ser-OH	KTTKSOH	57	9.3	563.64	564.45
2	Ac-Lys-Thr-Thr-Lys-Ser-OH	AcKTTKSOH	55	13.1	605.68	606.72
3	Lip-Lys-Thr-Thr-Lys-Ser-OH	LipKTTKSOH	47	20.9	751.95	753.09
4	Pal-Lys-Thr-Thr-Lys-Ser-OH	PalKTTKSOH	56	23.4	802.05	803.55
5	H-Lys-Thr-Thr-Lys-Ser-NH_2	KTTKSNH_2	62	11.4	562.66	563.17
6	Ac-Lys-Thr-Thr-Lys-Ser-NH_2	AcKTTKSNH_2	58	15.5	604.69	605.66
7	Lip-Lys-Thr-Thr-Lys-Ser-NH_2	LipKTTKSNH_2	42	22.8	750.97	752.12
8	Pal-Lys-Thr-Thr-Lys-Ser-NH_2	PalKTTKSNH_2	58	25.3	801.06	802.56
9	H-Lys-Thr-Thr-Arg-Ser-OH	KTTRSOH	58	8.9	591.65	592.69
10	Ac-Lys-Thr-Thr-Arg-Ser-OH	AcKTTRSOH	55	12.6	633.69	634.88
11	Lip-Lys-Thr-Thr-Arg-Ser-OH	LipKTTRSOH	52	19.2	779.96	781.02
12	Pal-Lys-Thr-Thr-Arg-Ser-OH	PalKTTRSOH	56	21.5	830.06	831.07
13	H-Lys-Thr-Thr-Arg-Ser-NH_2	KTTRSNH_2	63	11.0	590.67	591.77
14	Ac-Lys-Thr-Thr-Arg-Ser-NH_2	AcKTTRSNH_2	65	15.1	632.71	633.77
15	Lip-Lys-Thr-Thr-Arg-Ser-NH_2	LipKTTRSNH_2	54	23.9	778.98	779.65
16	Pal-Lys-Thr-Thr-Arg-Ser-NH_2	PalKTTRSNH_2	61	26.3	829.08	828.01
17	H-Arg-Thr-Thr-Arg-Ser-OH	RTTRSOH	68	8.3	619.67	620.61
18	Ac-Arg-Thr-Thr-Arg-Ser-OH	AcRTTRSOH	63	13.1	661.70	662.72
19	Lip-Arg-Thr-Thr-Arg-Ser-OH	LipRTTRSOH	61	22.5	807.98	808.52
20	Pal-Arg-Thr-Thr-Arg-Ser-OH	PalRTTRSOH	65	24.5	858.08	859.80
21	H-Arg-Thr-Thr-Arg-Ser-NH_2	RTTRSNH_2	65	10.2	618.69	619.58
22	Ac-Arg-Thr-Thr-Arg-Ser-NH_2	AcRTTRSNH_2	66	14.8	660.72	661.51
23	Lip-Arg-Thr-Thr-Arg-Ser-NH_2	LipRTTRSNH_2	53	23.1	806.99	807.23
24	Pal-Arg-Thr-Thr-Arg-Ser-NH_2	PalRTTRSNH_2	62	26.5	857.09	858.59
25	H-Arg-Thr-Thr-Lys-Ser-OH	RTTKSOH	59	8.8	591.65	592.54
26	Ac-Arg-Thr-Thr-Lys-Ser-OH	AcRTTKSOH	58	12.4	633.69	634.78
27	Lip-Arg-Thr-Thr-Lys-Ser-OH	LipRTTKSOH	52	19.1	779.96	781.22
28	Pal-Arg-Thr-Thr-Lys-Ser-OH	PalRTTKSOH	58	21.8	830.06	830.98
29	H-Arg-Thr-Thr-Lys-Ser-NH_2	RTTKSNH_2	58	11.9	590.67	591.82
30	Ac-Arg-Thr-Thr-Lys-Ser- NH_2	AcRTTKSNH_2	54	15.5	632.71	633.73
31	Lip-Arg-Thr-Thr-Lys-Ser- NH_2	LipRTTKSNH_2	51	23.5	778.98	781.07
32	Pal-Arg-Thr-Thr-Lys-Ser- NH_2	PalRTTKSNH_2	59	26.1	729.86	730.53

K=Lys, T=Thr, S=Ser, R=Arg, Ac=acetyl, Lip=lipoyl, Pal=palmitoyl, e.g. PalRTTKSNH_2 = $C_{15}H_{31}$CO-Arg-Thr-Thr-Lys-Ser-NH_2.
